# From the teapot effect to tap-triggered self-wetting: a 3D self-driving sieve for whole blood filtration

**DOI:** 10.1038/s41378-023-00490-7

**Published:** 2023-03-21

**Authors:** Yuang Li, Xue Li, Lina Zhang, Xiaofeng Luan, Jiahong Jiang, Lingqian Zhang, Mingxiao Li, Jinghui Wang, Jiangang Duan, Haiping Zhao, Yang Zhao, Chengjun Huang

**Affiliations:** 1 Institute of Microelectronics of the Chinese Academy of Sciences, Beijing, 100029 China; 2 University of Chinese Academy of Sciences, Beijing, 100049 China; 3 Institute of Cerebrovascular Disease Research, Xuanwu Hospital of Capital Medical University, Beijing, 100053 China; 4 Department of Cellular and Molecular Biology, Beijing Chest Hospital, Capital Medical University / Beijing Tuberculosis and Thoracic Tumor Research Institute, Beijing, 101149 China

**Keywords:** Materials science, Chemistry, Engineering

## Abstract

Achieving passive microparticle filtration with micropore membranes is challenging due to the capillary pinning effect of the membranes. Inspired by the teapot effect that occurs when liquid (tea) is poured from a teapot spout, we proposed a tap-triggered self-wetting strategy and utilized the method with a 3D sieve to filter rare cells. First, a 3D-printed polymer tap-trigger microstructure was implemented. As a result, the 3 µm micropore membrane gating threshold (the pressure needed to open the micropores) was lowered from above 3000 to 80 Pa by the tap-trigger microstructure that facilated the liquid leakage and spreading to self-wet more membrane area in a positive feedback loop. Then, we implemented a 3D cone-shaped cell sieve with tap-trigger microstructures. Driven by gravity, the sieve performed at a high throughput above 20 mL/min (DPBS), while the micropore size and porosity were 3 µm and 14.1%, respectively. We further filtered leukocytes from whole blood samples with the proposed new 3D sieve, and the method was compared with the traditional method of leukocyte isolation by chemically removing red blood cells. The device exhibited comparable leukocyte purity but a higher platelet removal rate and lower leukocyte simulation level, facilitating downstream single-cell analysis. The key results indicated that the tap-triggered self-wetting strategy could significantly improve the performance of passive microparticle filtration.

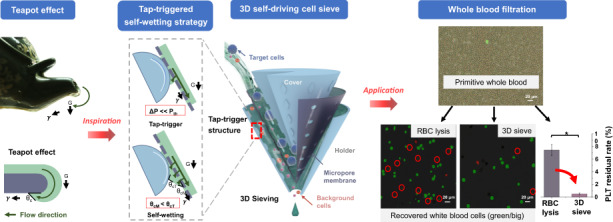

## Introduction

Microparticle separations with micropore membranes have been widely used in emerging applications, which range from environmental plastic microparticle filtration^1–3^ to biomedical cell filtration^4–7^. However, when the micropore diameter is scaled down to the 1 μm level, the well-known capillary pinning effect caused by liquid surface tension becomes more severe. This significantly lowers the performance of the membrane for filtration^8,9^. Theoretically, with a plain horizontal micropore membrane, the liquid should be pinned by the Laplace pressure caused by the sudden expansion shape of the micropore mouths. An extra pressure (Δ*P*) beyond the gating threshold (*P*_th_) was needed to enlarge the liquid front’s surface from the area of holes, extending to the whole membrane to gather the liquid into droplets^10–12^. Moreover, a reduction in porosity was often accompanied by the micropores’ diameter scaling down to 1μm; therefore, the margin between the micropores increases too much to be extended and covered by the liquid front only through the liquid’s gravity-induced hydrostatic pressure.

Traditionally, high-speed centrifuges and pneumatic supplies are used as the external driving force to overcome the capillary pinning effect and accelerate the filtration in practical applications^13–15^; however, with these methods, centrifuges or pumps are needed, increasing the complexity and costs^9^. Even worse, in some biomedical applications, the cells may undergo large hydromechanical forces due to the high acceleration and impact generated by high-speed centrifugation, resulting in cell damage or even cell death^16–18^. These disadvantages have hindered the broader use of micropore membrane-based microparticle separation.

To overcome the abovementioned drawbacks, researchers have contributed several strategic approaches to lower the gating threshold of micropore membranes. One approach was to increase the porosity of the micropore membrane^19,20^. Nevertheless, higher porosity dramatically increased the difficulties for the membrane materials and microfabrication techniques, especially when the pore size reached 1 μm^21^. Another successful approach was to adjust the gating threshold of the pores by prefilling the micropores with low-surface-energy liquid, as reported in the theory of the “liquid-based gating mechanism”^22–24^. However, these studies still involve drawbacks, such as possible contamination or damage, as different low-surface-energy liquids had to be added.

**Fig. 1:**
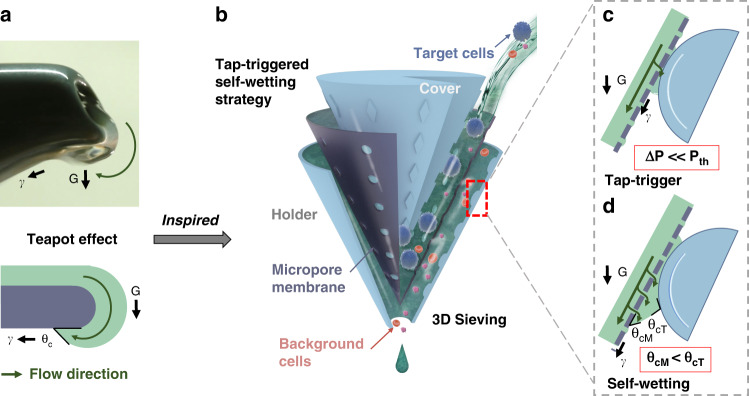
The teapot effect inspired the tap-triggered self-wetting strategy-based 3D cell sieving method. **a** The teapot effect and its physical model. **b** The tap-triggered self-wetting strategy-based 3D sieving method. **c** The tap-trigger microstructure drew liquid out from the underside of the inclined membrane. **d** The passed liquid flowed along the membrane to self-wet more of the membrane area, drawing more liquid flow through micropores in a positive feedback loop

As shown in Fig. 1, inspired by the teapot effect (Fig. 1a)^25^, we proposed a tap-triggered self-wetting strategy-based 3D sieving method (Fig. 1b) to address the gap. When pouring tea slowly, the liquid is absorbed and flows along the underside of the spout due to the hydrophilia of the underside surface. Then, the absorbed liquid might be pinned behind the capillary barrier, and this pinned liquid might spill over^26^. Likewise, we introduced a process called “tap-trigger” to cause the microscale fluid to permeate through the micropores and initially wet the inclined membrane’s small local region. Then the local self-wetting is broadcast to the entire membrane in a positive feedback loop, which is driven only by gravity (Fig. 1c). Furthermore, we introduced a process called self-wetting to bring the fluid from the upside to the underside of the membrane to merge the meniscus of the liquid front in micropores, helping the pinned liquid break the barrier at the mouths of the micropores (Fig. 1d).

To demonstrate the improvement in performance attained by the proposed method, in the following section, we introduced the “teapot effect” into the device design and 3D-printed a tap-trigger microstructure. The structure was placed under the inclined micropore membrane with the liquid on it to observe and evaluate the liquid behavior (Fig. 1c, d). Then, we implemented this tap-trigger microstructure into a 3D cone-shaped cell sieving device, as shown in Fig. 1b. The 3D cell sieve was further applied for leukocyte filtration from whole blood, in which the tap-triggered self-wetting strategy was evaluated.

## Results and discussion

### Principle of the tap-triggered self-wetting strategy

The tap-trigger process (Fig. 1c) was the essential precondition for further reducing the gating threshold of the entire micropore membrane. To achieve the tap-trigger process and draw liquid out from the underside of the membrane, first, the micropore membrane that tended to increase the hydrostatic pressure in the liquid was placed above the membrane, considering the dimension-raising strategy we previously proposed^27^. Then, after analyzing the micropores’ mechanical condition, we needed to locate an appropriate small local region to draw the microscale fluid and wet the membrane.

**Fig. 2:**
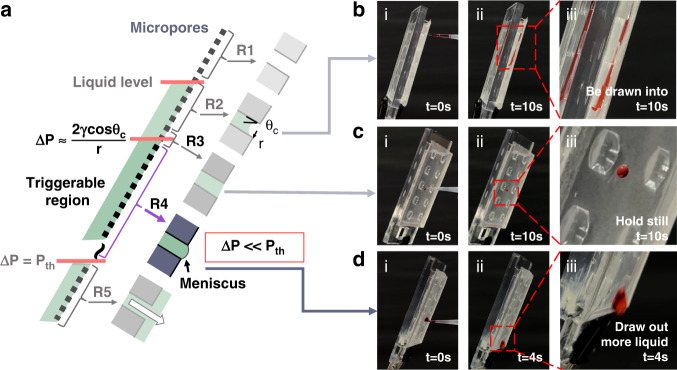
Descriptions and exploration results of the triggerable region on inclined micropore membranes for self-wetting. **a** Descriptions of different liquid states under the micropore membrane in five regions (R1–R5). **b**–**d** Observed different red ink droplet movements in the three regions: **b** R2, droplets were drawn into the membrane, **c** R3, droplets were held still, and **d** R4, more liquid was drawn by droplet out to flow along the membrane

To locate the appropriate position and increase the liquid depth, we divided the membrane into five regions (R1–R5) according to the relationship between the total pressure (Δ*P*) the liquid incurred and the gating threshold of the membrane (*P*_th_). As illustrated in Fig. 2a, the following regions were included. R1: no liquid; R2 and R3: where the surface tension dominated and the liquid front meniscus in the micropores was indented or flattened^28,29^; R4: where the hydrostatic pressure increased to form a liquid meniscus that protrude from the micropore mouths on the bottom surface of the membrane^30^, however, the main body of liquid front was still pinned by surface tension in the micropores^31^; and R5: where the hydrostatic pressure increased beyond the gating threshold (*P*_th_) of the micropores and pushed the liquid out^32^. The related fundamental mechanics and equations are described in the “fundamental mechanics” section in the Supplemental Material.

To achieve the tap-trigger process to self-wet the entire underside of the membrane, we further tapped a point in the triggerable region (Fig. 2a) with a finger-like structure. The structure’s surface contacts the protruded meniscus and draws out the liquid. After that, the liquid was gathered into a droplet to spread on the membrane’s bottom surface, driven by surface tension and gravity. Sequentially, the spreading of the liquid self-wetted the membrane and drew out more liquid to wet more area in a positive feedback loop. Once the micropores were broken through, the liquid would continuously draw out to self-wet the membrane and overcome the high gating threshold problem until the liquid became dry.

Notably, maintaining the spreading of the liquid on the bottom surface of the membrane rather than the trigger structure was essential for continuous self-wetting. Thus, we needed to ensure that the equilibrium contact angle (CA) on the bottom surface of the micropore membrane (*θ*_cM_) was less than that on the surface of the trigger structure (*θ*_cT_) (Fig. 1d). The CA at the thermodynamic steady state might not be equivalent to the spread ability but commonly dominated the spreading behavior of liquids on the smooth surface when CA was not small enough^33,34^. Finally, this strategy only needs gravity to provide hydrostatic pressure (Δ*P*), which is far smaller than the gating threshold (*P*_th_), to drain the liquid above the micropore membrane rapidly.

Accordingly, we verified the liquid state in different regions of the micropore membrane. Using red ink as a marker, we observed that when the red ink droplets were added to three different depths under the membrane (Fig. 2b-i, c-i, and d-i), the inside liquid pulled the droplets into the membrane (Fig. 2b-ii and iii), the droplets remained still (Fig. 2c-ii and iii), and more liquid was drawn out from the membrane (Fig. 2d-ii and iii). The results demonstrated the presence of different regions, and their locations match the corresponding description in Fig. 2a.

### Tap-triggered self-wetting microstructure characterization

**Fig. 3:**
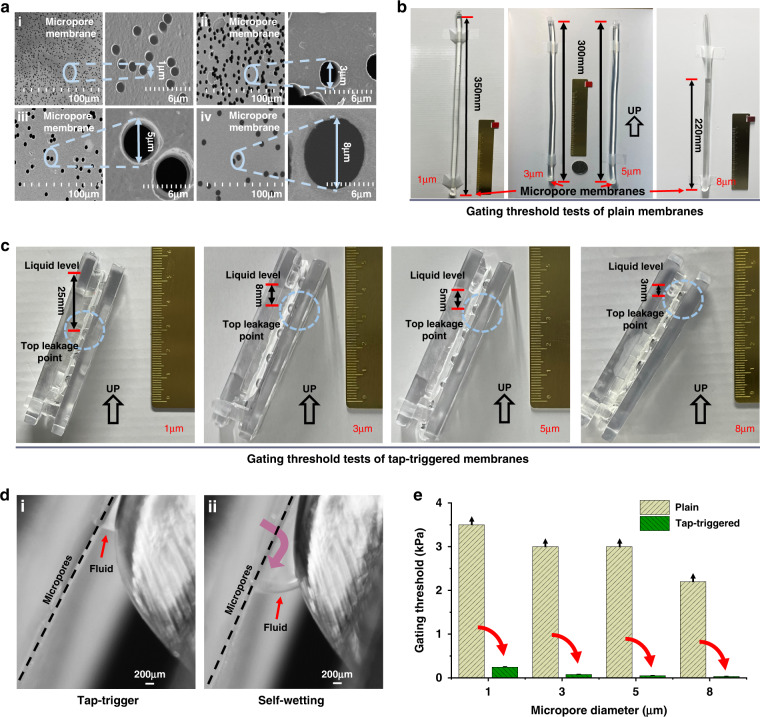
Gating threshold tests of the horizontal plain micropore membranes and the inclined membranes with tap-trigger microstructures. **a** SEM images of four types of membranes with (i) 1 μm, (ii) 3 μm, (iii) 5 μm and (iv) 8 μm micropores. **b** Liquid columns where the liquids could not break through the four types of plain horizontal micropore membranes mounted at the bottom end of the long hollow tube. **c** Gating threshold tests of the four types of membranes inclined and contacted with tap-trigger microstructures. **d** Photomicrographs of the self-wetting process after triggering: the structure initially helped the liquid to penetrate (i) and then drew more liquid out to self-wet more membrane area (ii). **e** Comparative analysis of the gating threshold between the horizontal plain membranes and tap-triggered inclined membranes with four sizes of micropores. The black arrows indicate that the actual gating thresholds of the plain membranes were beyond the measured values in impassable statuses

Before testing, scanning electron microscopy (SEM) images of the micropore membrane with four sizes of 1, 3, 5, and 8 μm were obtained (Fig. 3a). Moreover, the contact angles of the membrane and 3D-printed device surface were measured and recorded as 65.4° and 84.3°, respectively (Fig. S2). Both essential components satisfied the prerequisites.

Initially, plain membranes were horizontally assembled with different micropore sizes at the bottom end of the transparent hollow polymer tubes with inner diameters of 10 mm (Fig. 3b). The column height observed above the 2D micropore membrane was used to characterize the gating threshold of the plain micropore membrane. The liquid columns above the four types of membranes with the different-sized micropores placed at the bottom end of the hollow polymer tubes were prefilled and measured. After prefilling the liquid columns at depths of 350, 300, 300, and 220 mm above the membrane with 1, 3, 5, and 8 μm-sized micropores (Fig. 3a), we observed that all liquid columns were retained above the membranes (Fig. 3b). The filling processes for the four types of membranes were repeated three times, and no liquid draining through the membrane was observed in every test. According to the depths, we calculated the corresponding hydrostatic pressures, which were less than the gating threshold of the plain membranes.

To significantly reduce the gating threshold of the membranes, we proposed a tap-triggered structure and evaluated its trigger performance. Regarding the geometrical parameters of the tap-trigger structure, a set of comparative studies with three tap-trigger structures with different sizes and shapes was performed, and the results are provided in Fig. S3 (Supporting information). As observed from the obtained results, the structure with the shape of a hemisphere and a radius of 2 mm performed better than the other tested structures with other shapes or smaller sizes. These results might be attributed to the requirement for enough contact area between the trigger structure and the microporous membrane. In this configuration, the tap trigger structure could obtain sufficient gravity from the collected liquid, which moved out from micropores to overcome the adhesive force of the membrane and flow down. With the tap-trigger structures, we observed the liquid column depths, which were defined by the height difference between the liquid level and the top liquid leakage point, and recorded them as 24.4 ± 1.24, 7.6 ± 0.80, 5.0 ± 0.58, and 3.1 ± 0.75 mm for membranes with 1, 3, 5, and 8 μm micropores, respectively (Fig. 3c). Compared to the same plain membrane, with the tap-trigger microstructures, the gating thresholds of the membrane with 3 and 5 μm micropores were reduced from above 3000 to 80 Pa by approximately 35-fold and from above 3000 to 50 Pa by approximately 60-fold.

After being triggered, liquid self-wetting phenomena were observed (Fig. 3d) and were consistent with the designed expectation (Fig. 1c, d). In the process, the liquid initially penetrated the membrane triggered by the tap-trigger microstructure (Fig. 3d-i) and then continuously self-wetted more of the membrane area to draw more liquid through the micropores in a positive feedback loop (Fig. 3d-ii).

The comparative results of the gating threshold of four types of membranes with 1, 3, 5, and 8 μm micropores are shown in Fig. 3e, verifying that the proposed tap trigger microstructure could successfully lower the gating threshold and facilitate liquid drainage driven only by gravity.

### Hydrodynamics behavior of the 3D sieve

**Fig. 4:**
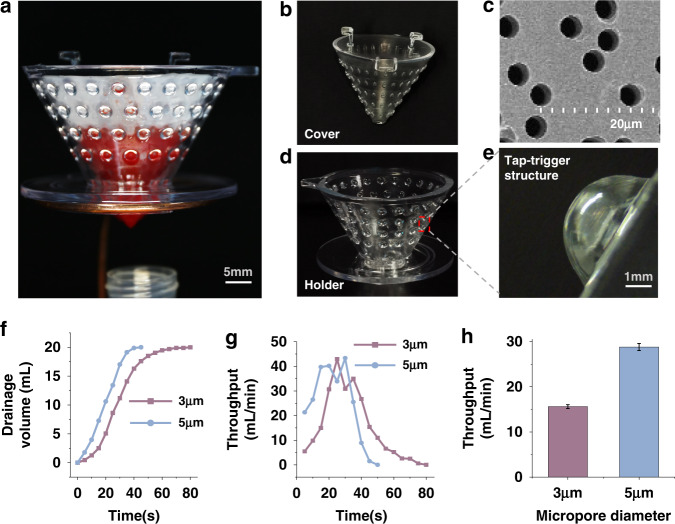
High-throughput performance of the tap-trigger self-wetting strategy-based 3D cell sieving device. **a** The photo of the assembled 3D sieve. **b** The photo of the 3D-printed conical channel structure. **c** The SEM image of micropore membranes with 3 μm micropores. **d** The 3D-printed holder **e** with the tap-trigger microstructure. **f** The drainage volumes and **g** transient throughputs of 20 mL DPBS draining from a 3D sieve with 3 and 5 μm-sized micropores, respectively, and **h** the average throughputs of 3 repeated tests, noted as the means ± SEMs

The fabricated 3D sieve is shown in Fig. 4, which was composed of a 3D-printed cone-shaped cover (Fig. 4b), a cone-shaped micropore membrane (Fig. 4c), and a substrate holder (Fig. 4d) equipped with tap-trigger microstructures (Fig. 4e) to touch the membrane.

To evaluate the device’s throughput, we used a precision balance to weigh the liquid draining over time and then translated weights to volumes (Fig. S4). We observed that 20 mL of Dulbecco’s phosphate-buffered saline (DPBS) drained out within 80 or 45 s in the micropore membrane filtration tests for 3- or 5 μm-sized micropore membranes, respectively (Fig. 4f). Figure 4g shows that the maximum transient throughputs for 3 and 5 μm micropores could reach more than 40 mL/min during a 20 mL DPBS drainage process. Figure 4h shows that the average throughputs of 3 and 5 μm micropore membrane filtration were 16 mL/min and 29 mL/min, respectively.

These experimental results suggest that compared to conventional micropore membrane filters, the proposed sieving device is more effective. The above results indicated that the 3D sieve could enrich target cells rapidly and effectively. In particular, the 3D sieve significantly improved the throughput compared to the reported filtration methods^19,20^. Since it was challenging to achieve passive filtration when the micropore size scaled down to 3 μm according to our previous work, here, we compared the throughput with the methods that used larger micropores for filtering and some of them assisted external drivers. Despite that, the passive 3D sieve’s transient throughput of approximately 40 mL/min was much higher than that of the typical throughput (below 1 mL/min) for the conventional micropore membrane filters, such as silicon-based plain filter (5 μm micropores, ~2.3 mL/min, driven with pump)^35^, track etched pore membrane (7.5 μm micropores, ~2 mL/min, driven with vacuum)^36^, SU-8 membrane (8 μm micropores, 5 mL/min, driven with pump)^37^, and PEGDA conical hole membrane (5.5 μm micropores, 0.2 mL/min, driven with pump)^38^. With the assistance of the teapot effect, our tap-triggered-aided 3D sieve with a smaller micropore size achieved throughput comparable to our previously proposed filter (8 μm micropores)^27^, and Wang et al. reported a 2.5-dimensional Parylene C micropore array with a large area and a high porosity (8 μm micropores)^3^.

Notably, this study’s primary efforts were focused on implementing the teapot effect into the micropore membrane-based filter and proposing the new tap-trigger structure. By applying this strategy, the capillary pinning drawback, which usually occurs in conventional micropore membrane filters, was well overcome, and a better filtering performance was achieved. Furthermore, possibilities still remain to further optimize the conical 3D sieve shape. For example, Yang et al. reported chemical-mechanical capillarity to design and generate an optimized shape, which made liquids slide off the surface very quickly. Similarly, we may shape the 3D sieve to follow the brachistochrone curve in our future study and further improve the filtration performance^39^.

### Leukocyte filtration by the 3D sieve

**Fig. 5:**
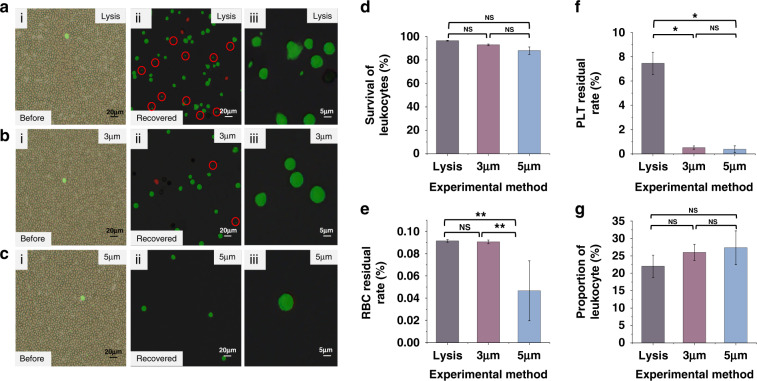
Performance results of the leukocyte preparation methods. **a**–**c** Microphotographs of cell samples before treatment (i) and recovered after RBC lysis, 3 and 5 μm micropore membrane-based filtration operations (ii, iii). **d** The proportion of surviving cells in leukocytes after cell sample preparation. **e** RBC residual rate and **f** PLT residual rate in cell samples after preparation. **g** The proportion of leukocytes after cell sample preparation. The datas are shown as the means ± SEMs and compared by unpaired Student’s t test or one-way analysis of variance (ANOVA). *P < 0.05, **P < 0.01; NS: no significance

In biomedical and clinical applications, an essential blood processing operation is filtering leukocyte from whole peripheral blood by removing red blood cells (RBCs) and platelets (PLTs). In this study, leukocyte filtration from whole blood was completed with the fabricated 3D cell sieving device with 3 and 5 µm micropores, and the performance of the method was evaluated. The results are shown in Fig. 5. Figure 5a–c shows merged brightfield (BF) and fluorescence field (FF) images of cells after different leukocyte enrichment methods, in which calcein AM-labeled live leukocytes are shown in green, and propidium iodide (PI)-labeled dead leukocytes are shown in red. In addition, the smaller green dots with a diameter of less than 5 μm labeled in the red circles (Fig. 5a-ii) were suspected as PLTs or cell debris (labeled with Calcein AM)^40,41^ and were visibly distinguishable by size.

Before leukocyte filtration, as shown in Fig. 5a–c-i, the proportion of leukocytes was deficient, and only a few green leukocytes could be observed (almost 0.1%) in whole blood cells. After leukocyte filtration, as shown in Fig. 5a–c-ii, the RBCs and PLTs were removed clearly by the 3D sieve, and the proportion of the resided was comparable with the leukocytes. The removal rates of RBCs using the 3 μm (Fig. 5a-ii) and 5 μm micropores (Fig. 5b-ii) are almost identical to that of RBC lysis (Fig. 5c-ii). Additionally, the removal rates of PLT are comparable. We noticed that fewer cells were counted after filtration using 5 μm-sized micropores than 3 μm-sized micropores, indicating that 3 μm-sized micropores were more suitable for leukocyte purification. Cell damage was evaluated by observing the detailed cell morphology. The detailed morphology of cells sieving by 3 μm (Fig. 5b-iii) and 5 μm micropore membranes (Fig. 5c-iii) seems intact. In contrast, the traditional chemical lysis method (Fig. 5a-iii) seemed to damaged the leukocytes, which might be attributed to the changes in osmotic pressure and the process of multiple centrifugations. Figure 5d shows that high viability rates of leukocytes (approximately 90%) were achieved by both 3D sieving and chemical lysis methods.

The hematology analyzer was used to count the proportions of leukocytes, RBCs, and PLTs before and after leukocyte filtration, and the results are shown in Fig. 5d–f, respectively. The RBC residual rates of the three methods were all less than 0.1% (Fig. 5e). Notably, the PLT residual rates of the 3D sieving methods were 0.52 ± 0.13% (3 μm) and 0.39 ± 0.27% (5 μm), which were significantly less than that of the lysis method (7.45 ± 0.92%, as shown in Fig. 5f). This result might have occurred because the centrifugation operation in RBC lysis cannot effectively remove PLTs, whereas the sieving process can remove the residual PLTs clearly to prevent subsequent tests from being impacted. As shown in Fig. 5g, the leukocyte proportions of 3D sieving methods were 27% (5 μm) and 26% (3 μm), slightly higher than lysis’s 22%. Furthermore, our 3D cell sieving method showed the advantage of the better PLT removal capability, which may overcome PLT contamination, which is commonplace during leukocyte preparations^42^.

### Cell quality characterization of enriched leukocytes by flow cytometry

**Fig. 6:**
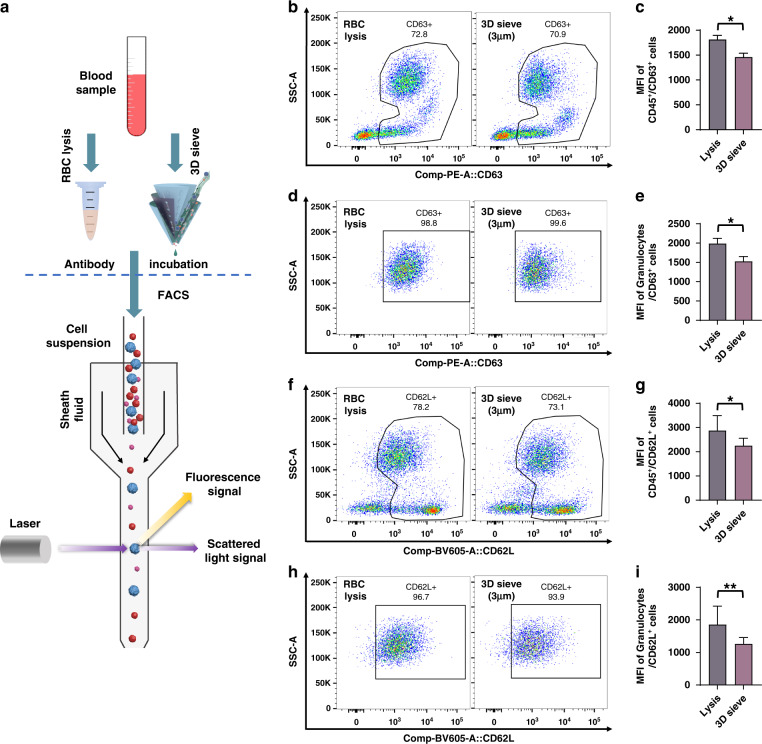
Cell quality of the leukocyte samples. **a** Schematic of the operation flow of the experiment. Regional plots and statistical plots of the CD63 expression in Leukocytes **b, c** and Granulocytes **d, e**. Regional plots and statistical plots of CD62L expression in Leukocytes **f, g** and Granulocytes **h, i**. The statistical datas are shown as the means ± SEMs and compared by unpaired Student’s t test. *P < 0.05, **P < 0.01

Furthermore, a low simulation level was crucial for immune cell filtration to accurately analyze immune mechanisms that are related to diseases^43–45^. We assessed the cellular quality of enriched leukocytes labeled with CD45, CD62L, and CD63 fluorescent antibodies by flow cytometry (Fig. 6a), following the cell manipulation and circle gating strategies shown in Fig. S5. CD63 is among the markers of neutrophil degranulation, and the mean fluorescence intensities (MFIs) of CD63 were used here to mark the stimulation of leukocytes^46^. The MFIs of CD63 in total leukocytes were initially compared (Fig. 6b), and the results showed that the MFI of CD63 in the lysed leukocytes was higher than that in the 3D sieved leukocytes (Fig. 6c). These results demonstrated that 3D sieving based on the self-wetting strategy could effectively reduce the stimulation rate of leukocytes.

CD62L is a vital adhesion molecule expressed by circulating neutrophils during adhesion^47,48^. Leukocyte activation and adhesion to endothelial cells are critical factors in the development of inflammation-related diseases^49^. Similarly, the MFIs of CD62L for total leukocytes were compared between the two methods (Fig. 6f), and the results showed that the MFI of CD62L-labeled leukocytes after lysis was significantly higher than that of the sieved cell samples (Fig. 6g), suggesting that cell stimulation might be avoided by our method. Then, the MFIs of CD62L granulocytes in the cell samples were compared (Fig. 6h), and the results showed that the MFI of CD62L of the granulocytes purified by the chemical method was significantly higher than that of granulocytes purified by our method (Fig. 6i).

The above results indicate that our 3D sieving method, which was developed based on a self-wetting strategy, could significantly lower the stimulation level of leukocytes and maintain cell homeostasis compared with the traditional chemical lysis method.

## Conclusion

In this paper, inspired by the teapot effect, we proposed a tap-triggered self-wetting strategy and further implemented this strategy in a 3D sieve for leukocyte filtration from whole blood. By introducing this strategy, the gating threshold of the 3 μm-sized micropore membrane was successfully lowered to 2.7%. The implemented 3D sieve with tap-trigger microstructures showed a higher performance for leukocyte filtration from whole blood than that of the traditional chemical RBC lysis method, indicating its potential for high-efficiency cell filtration in biomedical applications.

## Materials and methods

### Materials and data analysis

In this study, the relevant materials used for cell culture and processing in the experiment included BSA (37525, Thermo Fisher, USA), DPBS (21-321-CV, CORNING, USA), ACK Lysing Buffer (A10492-01, Gibco, Thermo Fisher, USA), and Flow Cytometry Staining Buffer (TNB-4222-L500, TONBO Biosciences, USA). APC anti-human CD45 (APC-65064, Proteintech, USA), Brilliant Violet 605 anti-human CD62L (304834, Biolegend, USA), and PE anti-human CD63 (353004, Biolegend, USA) were used to measure the quality of the target cells. Calcein-AM (C3099, Thermo Fisher, USA) and PI (P3566, Thermo Fisher, USA) were used as indicators of cell activity and cell state.

The micropore membrane was composed of polyethylene terephthalate (PET) material and purchased from the Heavy Ion Research Facility in Lanzhou (HIRFL), China. The thicknesses of the membranes with 8, 5, 3, and 1 μm micropores were 22, 12, 27, and 11 μm, respectively; correspondingly, their porosities were 10.1 and 11.8% and 14.1 and 11.8%, respectively.

During the testing and counting of the 3D sieve with whole blood cells, ImageJ software, as recommended by the National Institutes of Health (NIH), was used to enumerate cells on the BF and FF images. When processing streaming data, Flowjo software was used to analyze the results. IBM SPSS Statistics software was used to analyze variance. All data in this study are expressed as the mean ± SEM from no less than three independent assays.

### Characterization of the tap-trigger microstructure’s hydrodynamics behavior

Before evaluating the tap-trigger microstructure device, simple characterization experiments were performed using ink to define the positions of the five regions mentioned in Fig. 2a on the inclined micropore membrane. The shape and flow state of the ink droplets at different depth positions were observed to evaluate the pressure.

For comparison, we initially horizontally assembled plain membranes with different micropore sizes at the bottom end of the transparent hollow polymer tubes with inner diameters of 10 mm. Likewise, the liquid column height observed above the 2D micropore membrane was used to characterize the gating threshold of the plain micropore membrane.

To better understand the working principle of the tap-trigger microstructure, we then explored the hydrodynamic performances of tap-trigger microstructures with 1, 3, 5, and 8 μm micropores. Typically, a column of 3D-printed (Lite 300, UnionTech, China) tap-trigger microstructure was assembled to touch the bottom surface of micropore membranes. A horizontal microscope was used to observe and record the detailed process. With this setup, we slightly added liquid above the membrane to raise the liquid column and measured the highest liquid column when the liquid started leaking from the trigger point. The liquid columns with the four types of membranes with different pore sizes were recorded, converted to hydrostatic pressures, and further compared with the plain membranes to characterize the lowering effect of the gating threshold by the device.

All above operations were repeated at least three times, and all tested micropore membranes were used as purchased without any further surface treatments.

### Designing and fabricating the 3D cell sieving device

As shown in Fig. 1b, from inside to outside, the 3D cell sieving device was composed of a cone-shaped cover, a micropore membrane, and a substrate holder in a sandwich configuration. The cover, with a height of 50 mm and a cross-section cone angle of 60 degrees, was designed to shape the liquid above the membrane into a thin film between the membrane and tightly constrict the holder’s membrane. Lines of diamond-shaped structures with a height of 2 mm were designed to be placed on the cone’s bottom surface to support the cover. The tap-trigger microstructures with a height of 2 mm and plane radius of 2 mm were designed to radially arrange on the substrate holder’s top surface to touch the membrane after assembly. The geometrical outline shape of the substrate holder was designed to match the cone-shaped cover. The cover and the holder were 3D-printed (Lite 300, UnionTech, China) with transparent resin material. The middle interlayer of the sieving device was a cone-shaped micropore membrane. A practical heat sealing and cutting procedure formed the 3D cone-shaped micropore membranes^27^.

After fabrication, the throughputs of the 3D sieve with 3 or 5 μm-sized micropores were quantified with a precise balance (Fig. S4) and repeated at least three times.

### Evaluation of leukocyte filtration capability from whole blood

To demonstrate the effect of the proposed tap-triggered self-wetting strategy for microparticle separation in biomedical applications, we used the fabricated 3D cell sieving device to separate leukocytes from whole blood. The volunteers from the research group donated all blood samples used in the experiment, and all volunteers provided informed consent. This study was approved by the Medical Ethics Committee of Xuanwu Hospital, Capital Medical University ([2019] 119). Ethylenediaminetetraacetic acid (EDTA) tubes were used to collect peripheral blood from healthy volunteers.

Considering the typical size distribution of leukocytes (>6 μm), while RBCs (5 μm) and PLTs (<3 μm) and the deformability of these microparticles, 3 and 5 μm micropore sizes of the micropore membrane were selected and compared for whole blood filtration. Before sieving, the micropore membranes were soaked in 1% BSA for surface protein modification to reduce cell damage and residue on the membrane surface.

Meanwhile, following the manufacturer’s instructions for available chemical RBC lysis and leukocyte isolation, another set of whole blood samples was treated to compare the resulting performance with our methods, including the residual rates of RBCs and PLTs, filtration, and quality of leukocytes. All operations were repeated at least three times.

### Evaluation of the filtered leukocyte quality

Fluorescence microscopy, hematology analysis, and flow cytometry were used to characterize the quality of recovered cells following standard protocols from the manufacturers. First, the target cell proportion and viability rate were analyzed. The cell dead/alive fluorescence indicator Calcein AM/PI was used to stain the cell samples of the primary whole blood cells and the recovered cells by the three methods. After incubation (37 °C, 5% CO_2_) for 15 min, the target cells were photographed under a fluorescence microscope (IX-73, Olympus, Japan) and then counted.

Second, routine complete blood count (CBC) analysis was performed on a hematology analyzer (Sysmex XN-2000/3000 automatic blood cell analyzer, Sysmex Corporation, Japan). Then, the residual rate of nontarget cells (RBCs or PLTs) and the recovery purity of leukocyte samples were calculated^50,51^. After the preparations were completed, the leukocyte samples and the primary whole blood samples were resuspended in 1 mL DPBS and then analyzed to calculate the proportion of leukocytes, RBCs, and PLTs.

Finally, the leukocyte samples were labeled with antibodies, and the cell quality was further analyzed using flow cytometry. Antibody calibration and cell sample processing are shown in Fig. S5. The average fluorescence intensities of the CD62L and CD63 antibodies were used to characterize the stimulation of leukocytes. The detailed procedure for cell analysis with flow cytometry is presented in the Supplemental Material.

## Supplementary information

A clean version of the Supporting Information
