# Supplementary material for: From the teapot effect to tap-triggered self-wetting: a 3D self-driving sieve for whole blood filtration
Source: Microsyst Nanoeng. 2023 Mar 21;9:30. doi: 10.1038/s41378-023-00490-7 (PMC10027851; doi:10.1038/s41378-023-00490-7)
Supplement: Supplementary file 1 — A clean version of the Supporting Information [file 41378_2023_490_MOESM1_ESM.docx]

**Supporting Information**

From teapot effect to tap-triggered self-wetting: a 3D self-driving sieve for whole blood filtration

Yuang Li^1,2^, Xue Li^3^, Lina Zhang^4^, Xiaofeng Luan^1,2^, Jiahong Jiang^1^, Lingqian Zhang^1^, Mingxiao Li^1^, Jinghui Wang^4^, Jiangang Duan^3^, Haiping Zhao^3,*^, Yang Zhao^1,*^, and Chengjun Huang^1,2,*^

1. Institute of Microelectronics of the Chinese Academy of Sciences, Beijing 100029, China

2. University of Chinese Academy of Sciences, Beijing 100049, China

3. Institute of Cerebrovascular Disease Research, Xuanwu Hospital of Capital Medical University, 45 Changchun Street, Beijing 100053, China.

4. Department of Cellular and Molecular Biology, Beijing Chest Hospital, Capital Medical University, Beijing Tuberculosis and Thoracic Tumor Research Institute, 101149, Beijing, China.

***Corresponding Author:** huangchengjun@ime.ac.cn; zhaoyang@ime.ac.cn; [zhaohaiping@xwhosp.org](mailto:zhaohaiping@xwhosp.org).

**Contents:**

1. Flow simulation and analysis of the structure

2. Fundamental mechanism

3. Verification of equilibrium contact angle

4. Operation of the whole blood cells

5. Construction and verification of the throughput

6. Experimental methods and principles of flow cytometry

**Flow simulation and analysis of the structure**

A simulation of the structure was performed. The first is for the diamond-shaped structure on the cone slope, as shown in Fig. S1a. The liquid diversion structure effectively increases the liquid flow rate, minimizes the cell residue on the vertebrae, and improves filtration effectiveness. Moreover, the three-dimensional strategy, the role of lateral flow, as shown in the simulation Fig. S1b, under the action of gravity to further reduce the filtrating dead zone while improving the use of microporous. Those results demonstrated that the device structure could be used for high-throughput filtration of liquids and sieving large-volume samples and blood.


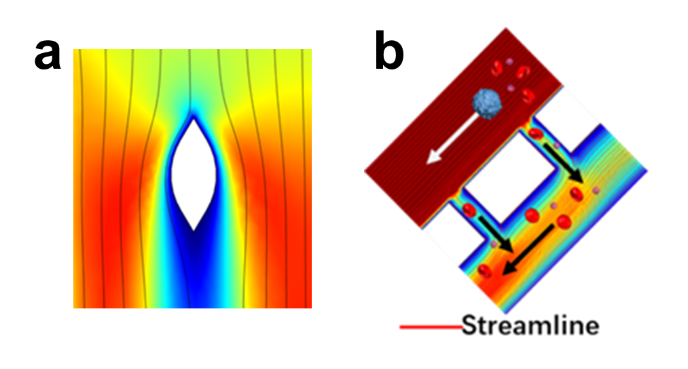


**Fig. S1** A simulation of the structure was performed. **a.** Simulation of liquid diversion with a diamond-shaped structure. **b.** Simulation of microporous lateral flow.

**Fundamental mechanism**

Initially, the fundamental mechanism behind the challenge and the strategy were rationally explained. At the micropores' underside mouths, the liquid front almost only suffered the surface tension because the hydrostatic pressure in the pores is negligibly slight. The Young–Laplace equation describes the surface tension (P) as follows:^1^

P=γ($\frac{1}{R1}+\frac{1}{R2})$ (1)

The pressure is larger on the concave side of the meniscus (gas–liquid interface) than on the convex side. Where γ is the surface tension factor of the liquid. R1 and R2 are the principal radii of curvature of the meniscus related to the contact angle and microchannel shape. When the liquid is in the hole, the state can be approximated as the liquid in the cylindrical microchannel. If the microchannel is a closed cylinder with radius r, the fluid boundary meniscus in the channel is relatively static, and the two principal radii of curvature of the meniscus are equal, substituting R = r/cosθ_c_ into Equation (1), the Laplace pressure of the cylindrical channel can be obtained as follows:

P=$\frac{2\gamma cos\theta c}{r}$ (2)

Therefore, when the driving pressure is insufficient, the liquid is bound inside the micropore to form a curved liquid surface (Figure 2a). Here we approximate the use of hydrostatic pressure to replace the driving force of the liquid in the hole, which is described as follows:

ΔP=ρgh (3)

According to the equation (2) and (3), the height of the liquid level at which the two forces are in equilibrium can be deduced as follows:

h =$\frac{2\gamma cos\theta c}{r\rho g}$ (4)

And when the liquid is under too much pressure to form the burst valve state, then the liquid pressure can be derived and results in a simple form expressed as:^2^

P_th_=$\frac{2\gamma sin\alpha}{r+x}$ (5)

Where x is the wetting length of the convex meniscus, and the meniscus shape is described by α = −θ_c_. A negative liquid pressure means a barrier for stopping liquid advancement. Based on the liquid level position and height (h), we divided the micropores into five zones, as shown in Figure 2a, and confirmed our conjecture using ink flow phenomena and lateral industrial microscopy.

**Verification of equilibrium contact angle**

A lateral industrial microscope was used to measure the contact angles of the membrane and 3D-printed devices' surface. The contact angles of the membrane and 3D-printed devices' surface were measured and recorded as 65.4° and 84.3°, respectively (Fig. S2). Both essential components satisfied the pre-requirements. It is worth noting that the difference in contact angle will be greater when on a 3D spherical trigger structure and membrane.


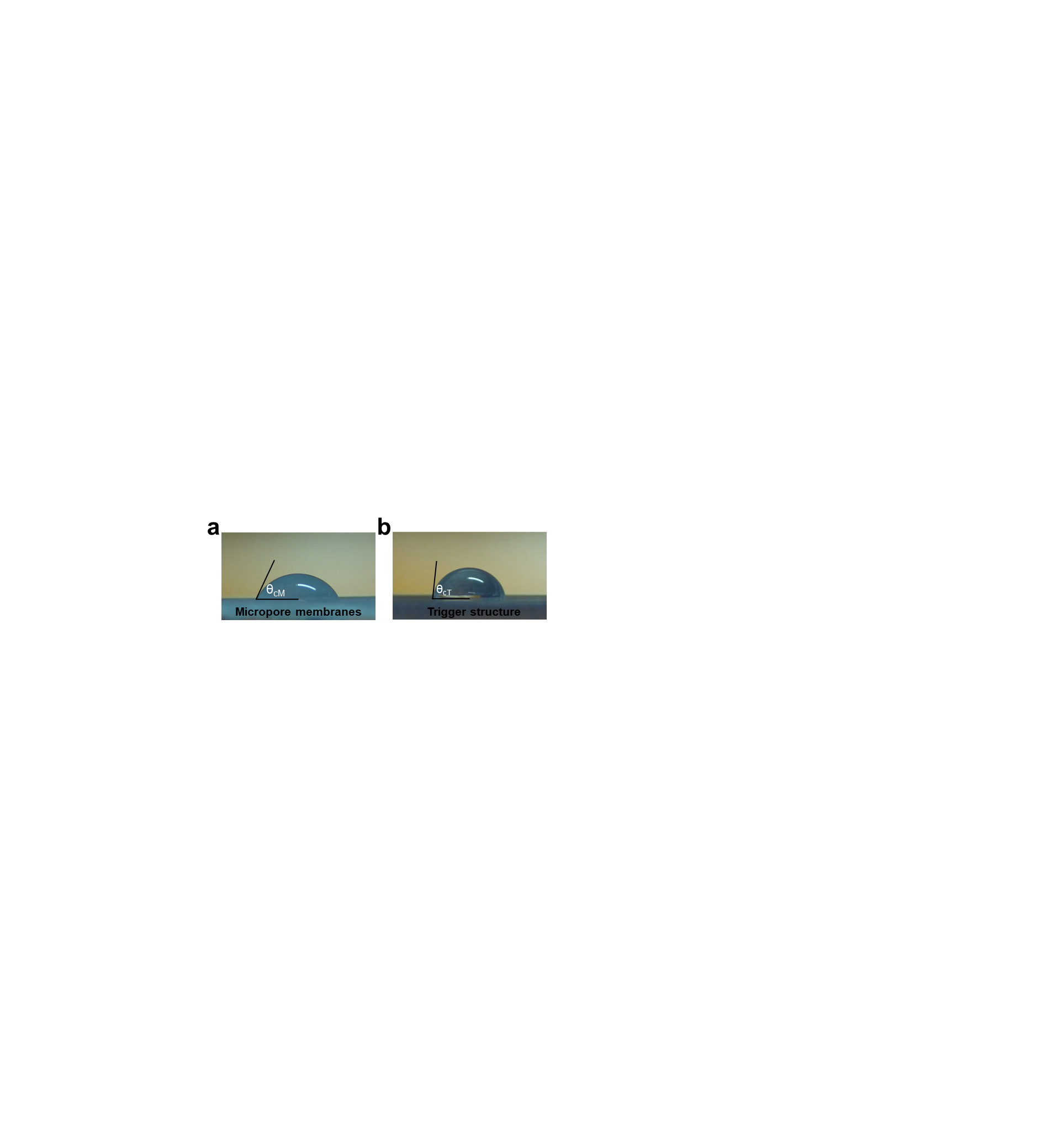


**Fig. S2** Measurements of contact angles on the micropore membrane and 3D-printed tap-trigger structure. **a.** The contact angle of the membrane. θ_cM_=65.4°. **b.** The contact angles of the 3D-printed devices' surface. θ_cT_=84.3°. θ_cM_<θ_cT_.

**Verify the effect of different sizes and shapes of tap-trigger structures**

To explore the better choice of trigger structure's critical geometrical parameters, we designed, 3D-printed, and evaluated the trigger performance of three different tap-trigger structures, which were a hemisphere structure with a radius of 2mm (Fig. S3a-i), a sphere structure with a radius of 1mm (Fig. S3a-ii), and a cone structure with both a radius and a height of 2mm (Fig. S3a-iii). The structures were assembled to contact the downside of the inclined micropore membranes with 3μm sized micropores, respectively. During the test, 2mL of deionized water was added to the chamber above the inclined membrane to measure the gating threshold and throughput. Then, we observed the liquid column's depths, which were defined by the height difference between the liquid level and the top liquid leakage point (Fig. S3b), and then translated to the gating thresholds. Here, TTS-x (tap-trigger structures) were used to label the structures shown in Fig. S3a-i, ii, and iii, respectively. Fig. S3c showed that the gating thresholds of membranes were reduced to 75±3.5Pa (TTS-1), 184±3.8Pa (TTS-2), and 186±2.4Pa (TTS-3) assisted with the three trigger structures, respectively. The results indicated that the TTS-1 achieved a lower gating threshold. We finally compared the time spent for drainage of 2mL deionized water assisted with the three structures, and the results (shown in Fig. S3d) indicated that the TTS-1 has the fastest trigger and filtration speed. Those results indicated that there is still enough room to improve the trigger and self-wetting performance by optimizing the tap-trigger structures' shape, size, surface topology, and other geometrical parameters.


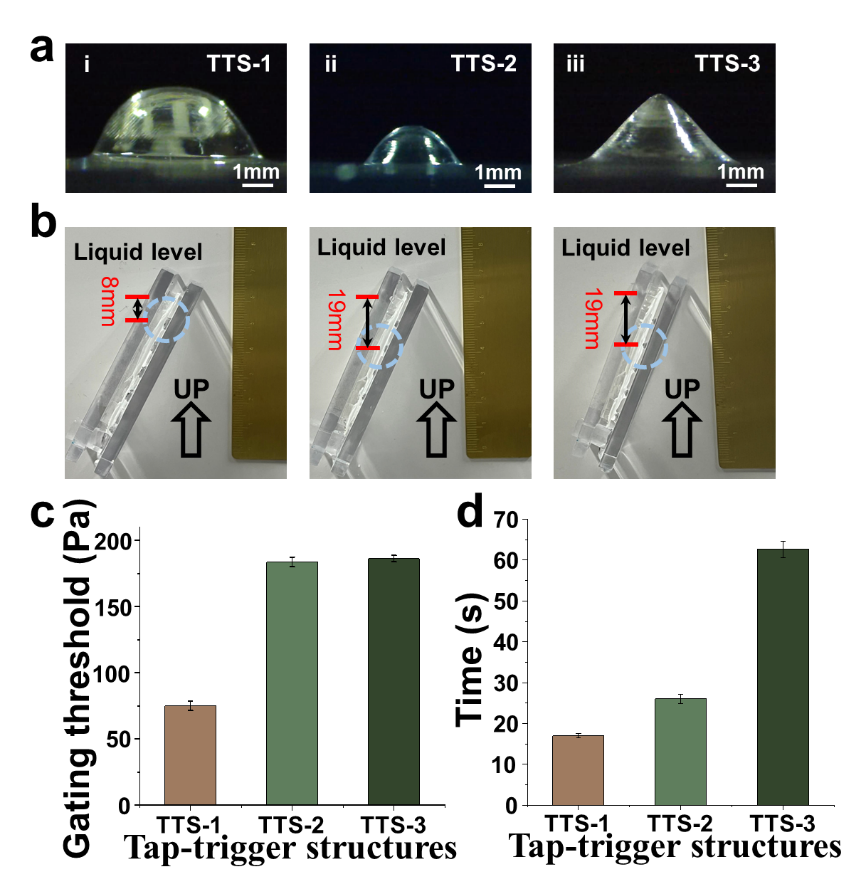


**Fig. S3** Critical geometrical parameters comparative experiments for evaluation of the trigger performance of three tap-trigger structures with different sizes and shapes. **a**. The photos of tap-trigger structures **b**. Gating threshold tests for three trigger structures with 3μm sized micropores. **c**. Comparative analysis of the gating threshold with three trigger structures. **d**. Compared the filtration time of 2mL with three structures.

**Operation of the whole blood cells**

Before the experiment, we equally divided the drawn blood sample to ensure that the blood volume for the initial manipulation experiment was 2mL. In the sieving and enrichment of blood, we used microporous membranes with pore sizes of 3μm and 5μm, respectively, to test the effect of pore size on the enrichment of leukocytes. For lysis operations, the ACK Lysing Buffer purchased from Gibco was used to extract leukocytes by removing the red blood cells from whole blood. The lysis process was carried out in strict accordance with the official instruction. Finally, the enriched cells were resuspended with 1mL DPBS buffer for subsequent analysis experiments. For the 3D cell sieving operation, based on the construction of the prototype device, sieving enrichment was performed as follows. A 2mL blood sample was diluted with 4mL DPBS and then poured into the sieving device. Then 4mL DPBS was used to wash the cone and micropore membrane surface and wait for sieving until the rest volume reached about 1mL. Finally, the 1mL DPBS of cell suspension was collected with a pipette for subsequent biological analysis experiments. After those operations, the collected samples were kept in a 4°C refrigerator and analyzed within an hour.

**Construction and verification of the throughput**

A high-precision balance was employed to weigh the liquid passed through the 3D sieving device in real-time, accompanied by a camera (an iPhone's built-in camera) and a timer (another iPhone's built-in application) used to record the video of weight changes and latency time. For microporous membranes of 3 and 5μm, every 20mL of DPBS was used as the initial volume in the tests for throughput quantification. The weight increase was then read out from the videos and divided by the density of DPBS to translate to the volume passed over time, that is, the real-time throughput.


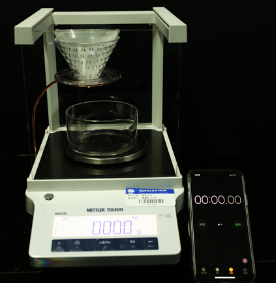


**Fig. S4** Setup of the test platform for measuring the 3D sieving device's throughput

**Experimental methods and principles of flow cytometry**

The flow cytometry sample manipulation and antibody modification process are shown in Fig. 6a. First, the cell samples obtained by the different methods were sorted and then centrifuged to obtain the cell precipitates. Then the cells were rinsed with 300μL Flow Cytometry Staining Buffer and centrifuged at 400xg for 5min at room temperature. Next, the desired antibody and flow cytometry staining buffer were mixed and added to the cells. And then, the cells were incubated at 4°C for 30min in darkness. After incubation, the cells were centrifuged at 400xg for 5min at room temperature. After that, the cell samples were washed with 300μL DPBS and centrifuged at room temperature for 5min. Finally, the samples were resuspended with 300μL DPBS for machine detection.

The strategy of the cell circle gate is shown in Fig. S5. The approximate regions of leukocytes were first circled according to the cell size distribution (Fig. S5a), after which the adherent cells were removed according to the signal intensity and cell size (Fig. S5b). The regions of the distribution of leukocytes can be further pinpointed using CD45 antibody labeling, while the region and ratio of granulocytes, monocytes, and lymphocytes can be obtained using the location of cell population distribution (Fig. S5c). Finally, as shown in Fig. S5d, the particular granulocyte regions were circled using CD45 and CD15 antibody labeling.


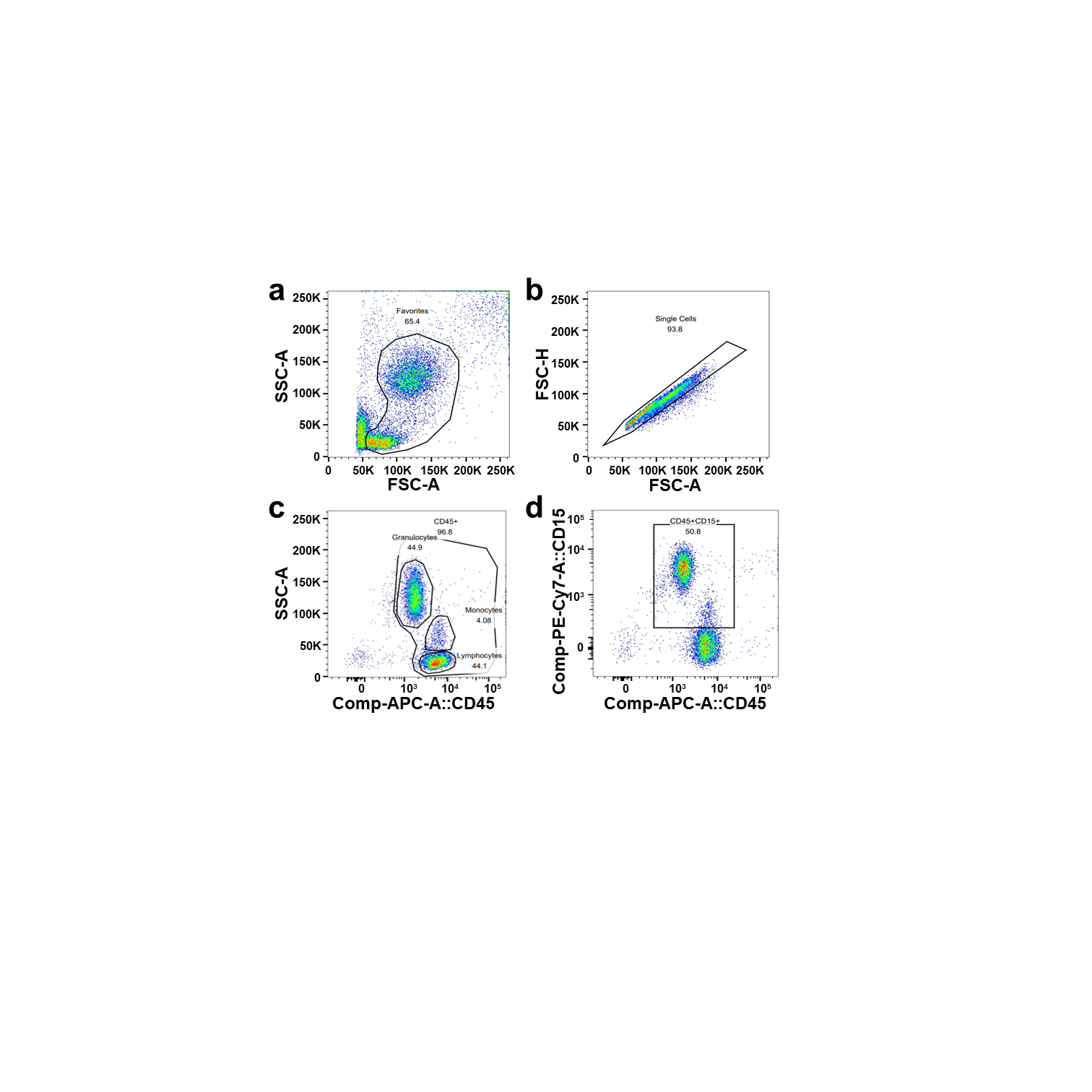


**Fig. S5** The strategy of the cell circle gate. **a.** Region of leukocyte distribution. **b.** Region of the single cells in leukocytes. **c.** Distribution regions of different cell types in leukocytes (labeled with CD45 antibody). **d.** Regions of granulocytes labeled with CD45 and CD15.

**REFERENCES**

1 Wang, S. *et al.* A Review of Capillary Pressure Control Valves in Microfluidics. *Biosensors (Basel)* **11**, doi:10.3390/bios11100405 (2021).

2 Chen, J. M., Chen, C.-Y. & Liu, C.-H. Pressure Barrier in an Axisymmetric Capillary Microchannel with Sudden Expansion. *Japanese Journal of Applied Physics* **47**, 1683-1689, doi:10.1143/jjap.47.1683 (2008).
